# Early Osteogenic Marker Expression in hMSCs Cultured onto Acid Etching-Derived Micro- and Nanotopography 3D-Printed Titanium Surfaces

**DOI:** 10.3390/ijms23137070

**Published:** 2022-06-25

**Authors:** Nora Bloise, Erik I. Waldorff, Giulia Montagna, Giovanna Bruni, Lorenzo Fassina, Samuel Fang, Nianli Zhang, Jiechao Jiang, James T. Ryaby, Livia Visai

**Affiliations:** 1 Department of Molecular Medicine (DMM), Center for Health Technologies (CHT), UdR INSTM, University of Pavia, 27100 Pavia, Italy; giulia.montagna04@universitadipavia.it; 2 Medicina Clinica-Specialistica, UOR5 Laboratorio di Nanotecnologie, ICS Maugeri, IRCCS, 27100 Pavia, Italy; 3 Research and Product Development, Orthofix Medical, Inc., 3451 Plano Parkway, Lewisville, TX 75056, USA; erikwaldorff@gmail.com (E.I.W.); samuel.fang@gmail.com (S.F.); nianlizhang@orthofix.com (N.Z.); jamesryaby@msn.com (J.T.R.); 4 Department of Electrical, Computer and Biomedical Engineering, Centre for Health Technologies (CHT), University of Pavia, 27100 Pavia, Italy; lorenzo.fassina@unipv.it; 5 C.S.G.I.-Department of Chemistry, Section of Physical Chemistry, University of Pavia, 27100 Pavia, Italy; giovanna.bruni@unipv.it; 6 Department of Material Science, University of Texas, Arlington, TX 76019, USA; jiangjc@uta.edu

**Keywords:** 3D-printed porous titanium implants, nanoscale topographies, acid etching processes, mesenchymal stem cells, osteogenic differentiation, Haralick texture analysis

## Abstract

Polyetheretherketone (PEEK) titanium composite (PTC) is a novel interbody fusion device that combines a PEEK core with titanium alloy (Ti6Al4V) endplates. The present study aimed to investigate the in vitro biological reactivity of human bone-marrow-derived mesenchymal stem cells (hBM-MSCs) to micro- and nanotopographies produced by an acid-etching process on the surface of 3D-printed PTC endplates. Optical profilometer and scanning electron microscopy were used to assess the surface roughness and identify the nano-features of etched or unetched PTC endplates, respectively. The viability, morphology and the expression of specific osteogenic markers were examined after 7 days of culture in the seeded cells. Haralick texture analysis was carried out on the unseeded endplates to correlate surface texture features to the biological data. The acid-etching process modified the surface roughness of the 3D-printed PTC endplates, creating micro- and nano-scale structures that significantly contributed to sustaining the viability of hBM-MSCs and triggering the expression of early osteogenic markers, such as alkaline phosphatase activity and bone-ECM protein production. Finally, the topography of 3D-printed PTC endplates influenced Haralick’s features, which in turn correlated with the expression of two osteogenic markers, osteopontin and osteocalcin. Overall, these data demonstrate that the acid-etching process of PTC endplates created a favourable environment for osteogenic differentiation of hBM-MSCs and may potentially have clinical benefit.

## 1. Introduction

Numerous types of bone fractures and diseases such as osteopenia, osteoporosis and spondylosis affect millions of people worldwide. Some minor bone damage can heal easily without surgery, thanks to the human body’s ability to self-renew and regenerate to lead the healing process. However, when surgery becomes mandatory to restore structural function (as in the case of knee replacement and spinal fusion) removal of damaged tissue and/or replacement with grafts is the approach usually taken by orthopaedists [1,2]. Autografts are still the most widely used bone grafts, followed by allografts, as both can guarantee osteogenesis, neovascularisation, osteoconductivity and osteoinductivity [3]. Nevertheless, they can have side effects and complications: donor site morbidity, bone tissue shortage, longer operative times, and a failure rate of up to 30% have been observed [4]. For these reasons, researchers are driven to find and design synthetic and more feasible solutions, such the development of polymeric biomaterials, 3D-bone implants and biodegradable devices [5,6,7,8]. Furthermore, great attention continues to be directed to the development of new technologies, such as surface modification techniques, to increase the actual clinical performance of implant devices [9,10]. Orthopaedic biomaterials are designed to be implanted in the human body as components of devices for replacing or repairing various tissues such as bone, cartilage or ligaments and tendons, and even to direct and guide bone restoration when necessary. In an interbody spinal fusion, the damaged intervertebral disc is removed and substituted with a bone graft material. The most widely used materials used for interbody cages include titanium and polyetheretherketone [11,12]. Titanium (Ti) and Ti alloys, such as titanium-6aluminium-4vanadium (Ti6Al4V), are largely used as implant materials as they provide mechanical support and have a decisive influence on the speed of osseointegration [13,14]. Despite its biocompatibility, the physiochemical properties of the surface topography of titanium have been considered essential for cell adhesion, cell proliferation, differentiation and implant osseointegration [15,16,17]. Previous studies have explored and demonstrated that micro/nano-structured surfaces on metal implants could have an enormous impact on the biological processes leading to implant osseointegration. To improve the interaction between the implant and stem cells, such as hBM-MSCs, several strategies have been exploited to modify titanium surfaces. Currently, popular techniques include additive (plasma spray, ion deposition, surface coating) or subtractive approaches (such as sandblasting and chemical etching) [18,19]. Acid-etching treatment, in combination with the shot-blasting approach, has been shown to increase the nanoroughness in the nanometre to submicron range in the titanium implant, thus improving the biological properties of the titanium implants and further enhancing their osseointegration [19,20,21]. Among the materials that have been suggested as an alternative to titanium, PEEK is a valid substitute for metal implants due to its stability and mechanical properties, similar to those of native bone tissue [22,23]. Thanks to these characteristics, PEEK has been widely used in orthopaedic and spinal surgery [24]. The bioinert properties of this material, however, hinder adhesion between the implant and surrounding tissue, which has led scientists to employ surface modification strategies such as sandblasting, plasma spraying, acid etching and micropatterning to modify the surface of PEEK to enhance its bioactivity [25]. Polyetheretherketone Titanium Composite is a new material technology, developed by Orthofix Medical Inc. (Lewisville, TX, USA), which is currently used in interbody spinal devices (CONSTRUX Mini PTC, Forza PTC and Pillar SA PTC) to restore the height of the spine and facilitate fusion in spinal fusion surgery [26]. This composite material combines two porous osteoconductive Ti endplates and a low-modulus radiolucent PEEK thermoplastic core. Compared to traditional devices made of monolithic PEEK material, porous osteoconductive Ti endplates that oppose bone may have the potential to better facilitate fusion by allowing the integration of bone into its three-dimensional microstructural pores. While the 3D structure of PTC endplates has previously been shown to enhance bone growth and osteogenic cell behaviour [12], the effect of the designed surface at the nano-scale has not been not examined. The current study aimed to examine the effect of the PTC endplate micro- and nanofeatures, produced by a proprietary etching process on the surface of titanium endplates. The manufacturing process of the porous Ti plates started with additive manufacturing (3D printing), followed by a series of proprietary post-printing processes, which included sandblasting, heat treatment and acid-etching. A preliminary study, limited in sample size and scope, showed that the manufacturing process of porous Ti plates creates a surface morphology at the micro and nano level that could be useful to facilitate casting (unpublished). Based on these preliminary data, the present work was developed. Specifically, the acid-etching process was isolated in the study group to investigate its effects on implant surface morphology at the micro- and nanoscale levels. hBM-MSCs are gaining increasing importance in the field of bone regenerative medicine due to their fundamental role in the bone healing and regeneration processes as precursors of osteoblasts [27,28,29]. To highlight the potential importance of micro- and nanofeatures in the overall performance of the new PTC device, we also examined the response of hBM-MSCs to these surfaces in terms of cell viability and expression of early osteogenic differentiation markers.

## 2. Results and Discussion

### 2.1. Surface Characterization

#### 2.1.1. Microscale Surface Morphology Characterization

**Figure 1:**
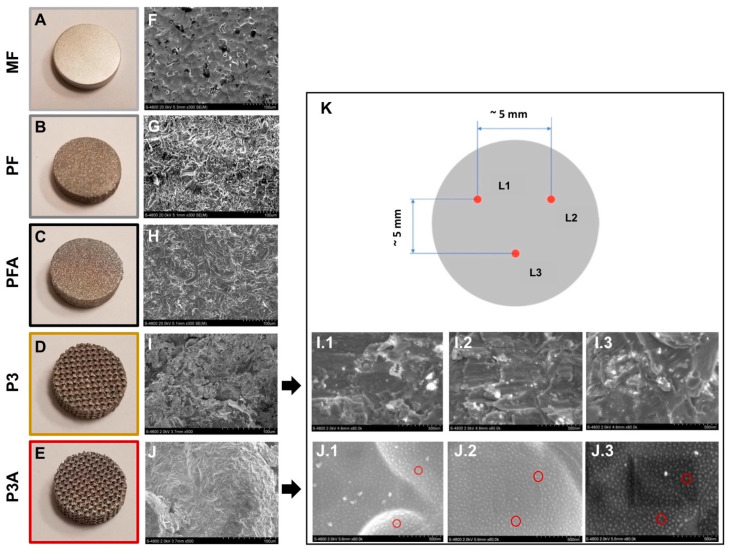
Ti6Al4V surface morphology. The right panel shows typical images of the different surfaces (**A**–**E**), while the middle panel shows their microscale SEM images (magnification: 300× for MF, PF and PFA and 500× for P3 and P3A, respectively) (**F**–**J**). In the right panel, representative secondary electron (SE) images were used to characterise the nanostructure of the porous samples, P3 and P3A. Images at 80 K× magnification were taken from both P3 (**I.1**, **I.2**, **I.3**) and P3A groups. In panel (**K**), schematic of image locations (indicated as L1, L2 and L3, respectively) for sample group P3 and P3A. Red circles in (**J.1**) indicate nanostructures with an average size of ~10 nm. An accelerating voltage of 2 kV was used.

Photographs of the sample groups are shown in Figure 1A–E. To characterise the differently modified surfaces, microscale surface morphologies were evaluated by SEM investigation (Figure 1F–J). SEM analysis revealed that MF (the machined sample) exhibited more flat features when compared to 3D-printed groups (PF and PFA), as expected (Figure 1F–H). The etched samples showed fewer irregularities and more rounded edges, which could be the result of acid etching. In addition, irregular texture in the form of small pits can be seen on the etched samples (Figure 1H), while these types of structure are less apparent on non-etched samples (Figure 1G). Regarding the 3D-printed porous samples, under high magnifications, the etched samples have more rounded edges than the non-etched samples because of the etching process (Figure 1I,J). To investigate the presence of nano-scale feature on porous samples, the surfaces of the porous non-etched sample and the porous etched sample were characterised in a Hitachi S-4800 field emission SEM at magnifications of 250,000× and above using an accelerating voltage of 2 kV and a working distance of 3.5 to 5.6 mm. The images at a magnification of 80,000× were taken for all 18 samples from P3 and P3A. For each sample, three images were taken at an approximate location as seen in Figure 1K. Figure 1(I.1–J.3) show all SEM images of samples from the porous non-etched and etched group at a magnification of 80,000×. The images taken from porous etched samples show the presence of nano-scale structures with an average size of ~10 nm on the surface, as indicated in Figure 1(J.1). However, this type of nano-scale structure is not apparent on porous non-etched samples (Figure 1(I.1–I.3)). Since the only difference in the process for both samples were the acid etching, it can be concluded that the acid etching process used for the PTC porous titanium endplate is capable of changing the material surface morphology and creating nano-scale structures on the surface.

#### 2.1.2. Microscale Roughness Measurement

**Figure 2:**
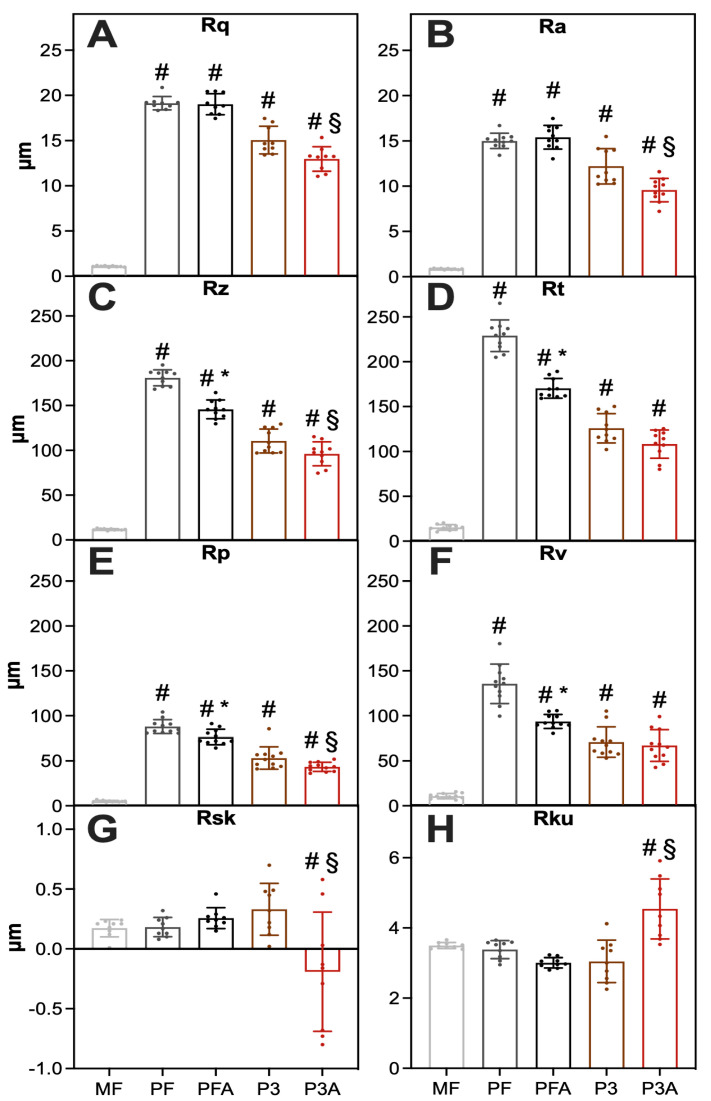
Measurement of roughness parameters on a microscale using an optical profilometer. Parameters evaluated: (**A**) mean roughness (Rq), (**B**) average roughness (Ra), (**C**) mean maximum profile height (Rz), (**D**) maximum profile height (Rt), (**E**) maximum profile peak height (Rp), (**F**) maximum profile valley depth (Rv), (**G**) Skewness (Rsk) and (**H**) Kurtosis (Rku). Statistical significance vs. MF was indicated by # (MF vs. other samples), while the symbol * means significant differences between PF and PFA (PF vs. PFA) and § between P3 and P3A (P3 vs. P3A).

Regarding the roughness measurements obtained by optical profiler, the machined control (MF) exhibited the lowest root-mean-squared roughness (Rq), roughness average (Ra), average maximum height (Rz), maximum profile height (Rt), maximum profile peak height (Rp) and maximum profile valley depth (Rv) when compared with the 3D-printed non-etched and etched samples (Figure 2A–F). However, the machined control sample showed Skewness (Rsk) and Kurtosis (Rku) values equal to the 3D-printed samples (Figure 2G–H). This indicated that the 3D-printed samples (both solid and porous groups) have a higher roughness (such as Rq, Ra, Rz, Rt, Rp, Rv) than the machined samples, and these data can fit with commonly accepted optimal parameters (Ra > 10 μm) [13,16]. Among the 3D-printed group, sample P3A presented the lowest Rq, Ra, Rz, Rt, Rp, Rv and Rsk (Figure 2A–G). The Rq and Ra levels were highest on the surface of the PFA samples (Figure 2A,B). The Rz, Rt, Rp and Rv were highest on the surface of PF samples (Figure 2C–F). When comparing etched (PFA and P3A) and non-etched groups (PF and P3), there was a general trend indicating lower Rq, Ra, Rz, Rt, Rp and Rv values for the etched groups (Figure 2A–F) compared to those of the non-etched group. This indicates that the etching process reduced the surface roughness of the samples as the acid etching process removed the sharp edges of the surface profile leading to a reduction in the surface roughness of the material at a microscale level.

### 2.2. hBM-MSCs Response to the Different Surface Morphologies

Enabling osteogenic differentiation by means of non-biochemical cues is a relevant topic in the field of bone tissue engineering, with important implications in the development of materials for inductive bone implants [30,31]. Numerous studies have shown that the topography and geometry of biomaterials could be a critical factor in promoting osteogenic differentiation of stem cells, such as hBM-MSCs, even without external chemical induction factors via mechanotransduction process [32,33]. To find out which of the examined surface structures influenced the behaviour of the cells, the response of hBM-MSCs was studied in terms of cell number, DNA content, ALP activity, bone-bound protein production and morphological analysis at day 7 of culture. All experiments were conducted in a culture medium without osteogenic supplements, such as dexamethasone, b-glycerophosphate and ascorbic acid, which are commonly added into media to promote osteogenic cell differentiation [34].

#### 2.2.1. Cell Viability and Morphology

**Figure 3:**
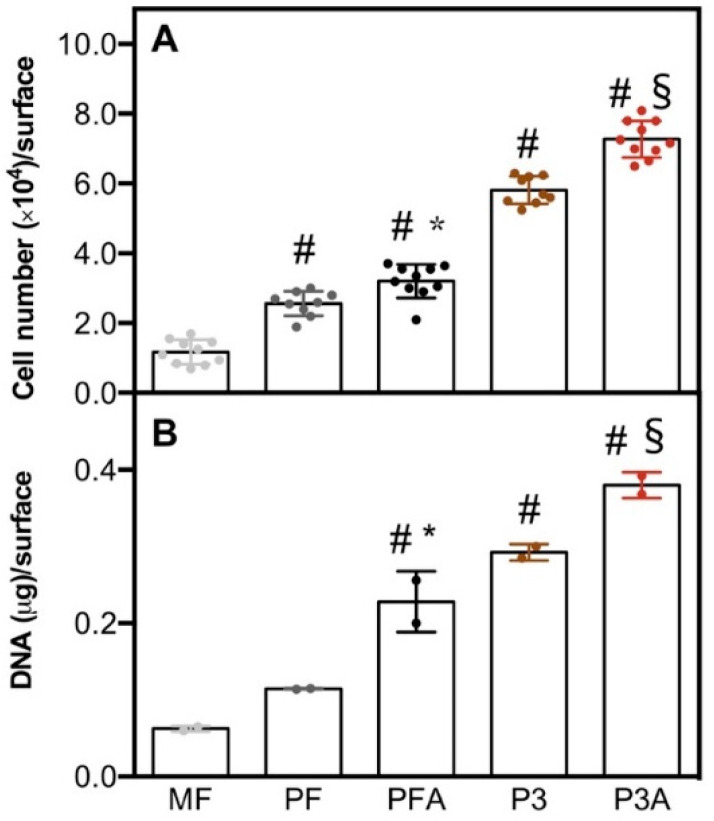
Cell number and DNA content. hBM-MSCs were cultured on each scaffold for 7 days and cell response was assessed by measuring the number of cells by the metabolic assay based on viability (**A**) and DNA content (**B**). Bars represent mean values ± standard deviation (SD). Statistical significance vs. MF was indicated by # (MF vs. other samples), while the symbol * indicates differences between PF and PFA (PF vs. PFA), and § between P3 and P3A (P3 vs. P3A).

hBM-MSCs viability was firstly analysed using a metabolic viability-based assay and through quantification of DNA content (Figure 3A,B, respectively). A higher number of cells was observed in the 3D-printed morphologies compared to the machined control (MF) (# *p* < 0.05, Figure 3A). Interestingly, the PFA surface showed a significant increase in the number of cells compared to the untreated PF (* *p* < 0.05). Similarly, there was a significant increase in the number of cells in P3A compared to the P3 surface (§ *p* < 0.05). These data were supported by the results of DNA quantification (Figure 3B). The DNA content on MF was significantly reduced compared to the other surfaces tested (# *p* < 0.05) and it was found that both acid-treated surfaces, PFA and P3A, significantly increased the DNA content of their untreated counterparts, PF and P3, respectively (* *p* < 0.05 and § *p* < 0.05) (Figure 3B).

**Figure 4:**
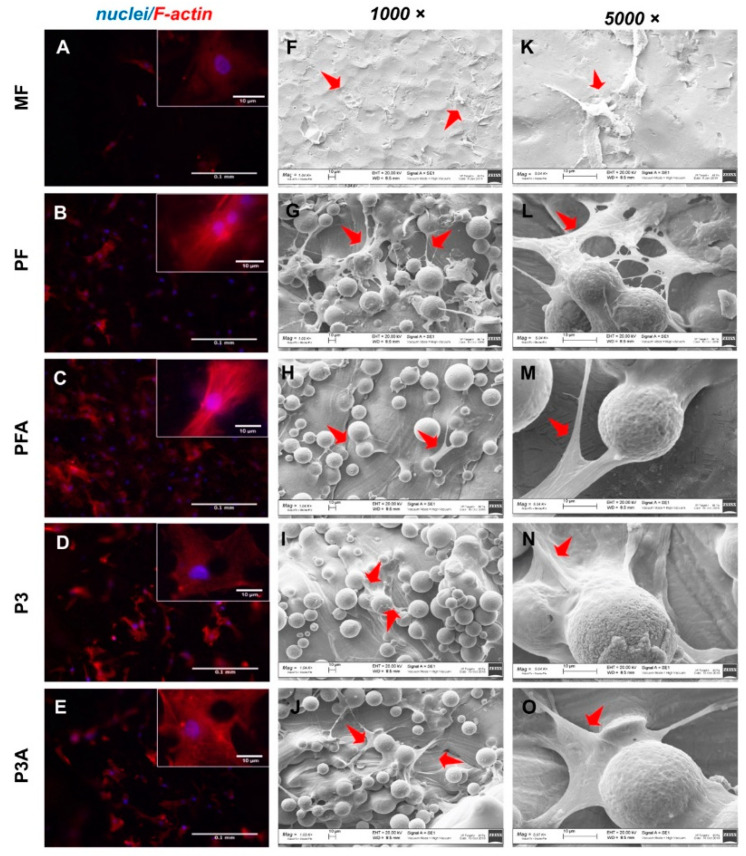
Cell morphology and distribution. The morphology of hBM-MSCs was examined by fluorescence microscopy (**A**–**E**) and scanning electron microscopy (SEM) (**F**–**O**) after 7 days of culture. The organisation of the cytoskeleton was observed after F-actin staining (red) and counterstaining of the nuclei (blue) at 10× magnification (scale bar: 100 µm). Magnified areas of the cells are shown in the insets (scale bar: 10 µm). Representative SEM images were collected at higher magnifications (1000× and 5000×). The red arrows show the filopodia-like extensions of the adhered cells on all tested surfaces.

Qualitative morphological investigation using microscopy is a valuable approach towards understanding stem cell behaviour, such as changes in the shape and morphology of cells once in contact with material [35]. Cells may assume different phenotypes according to surface roughness features [36]. It is broadly accepted that the nanoscale titanium surface stimulates the differentiation of various osteoblast lineages [37,38]. Furthermore, it was reported that osteogenic differentiation can be influenced by the initial morphology and cytoskeleton re-organization of hBM-MSCs at the nanoscale surface topography [39]. More, the acid-etching treatment of the titanium, by changing the micro-roughness of the material, improves the cell–titanium interaction, supporting better arrangement of the cytoskeleton and significantly accelerating the differentiation of stem cells towards the osteogenic lineage [38]. In this study, the morphology of hBM-MSCs was fluorescence microscopy of the red-labelled actin cytoskeleton and SEM at day 7 of culture. As shown in Figure 4, a different distribution pattern of actin stress fibres was observed between the different surfaces tested. Acidic stress fibres were almost absent in the MF surface (Figure 4A), while cells grown on 3D solid printed surfaces displayed an elongated spindle-shaped morphology with clearly visible actin stress fibres, mainly aligned parallel to the longitudinal axis of the cells within the whole cell body (Figure 4B,C). A different scenario was observed at the same time of culture onto porous samples: cells appeared with spindle-shaped morphology with abundant and highly organised actin stress fibres (Figure 4C,D). In addition, SEM investigation corroborated the cell viability and revealed a higher cell number on both solid (Figure 4G,M) and porous samples (Figure 4I,O) than on the MF control (Figure 4F,K). As was clearly visible in higher-magnification SEM images, cells were in the spreading phase in both solid and porous samples, with more cytoplasmic extensions and filopodial attachments and cellular protrusions (red arrows) extended throughout the material’s surfaces. By contrast, a round-shaped cell morphology was observed on the MF control (Figure 4F,K), suggesting a very low degree of adhesion on this surface.

#### 2.2.2. Osteogenic Markers

**Figure 5:**
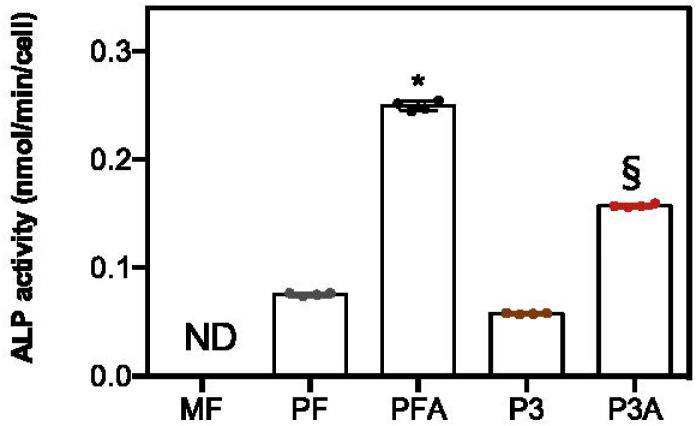
ALP activity. hBM-MSCs were cultured on each scaffold for 7 days and ALP activity was determined as described in the Materials and Methods section. ND = not detectable. Bars represent mean values ± SD. Symbol * indicates differences between PF and PFA (PF vs. PFA), and § between P3 and P3A (P3 vs. P3A).

Osteogenic differentiation can be studied through the assessment of alkaline phosphatase (ALP) activity and specific bone-related proteins. ALP activity is a very early marker of cellular differentiation of the osteogenic lineage [40]. The results obtained showed that ALP activity expressed as nmol/minute/cell was higher in cells grown on acid-treated surfaces than those grown on untreated ones, in both solid and porous groups. Indeed, a significant difference was obtained between PF and PFA (* *p* < 0.05) and then by comparing P3 to P3A (§ *p* < 0.05, Figure 5). In addition, ALP data were also confirmed when ALP activity was expressed as μmol/minute/μg of total protein content (Figure S1A) or as μmol/minute/μg of ALP (Figure S1B). It has previously been reported that ALP expression can increase with changes in surface microtopography, along with other proteins, such as bone morphogenetic proteins (BMPs), osteopontin and osteocalcin, which are all involved in the development of the bone tissue [41].

**Figure 6:**
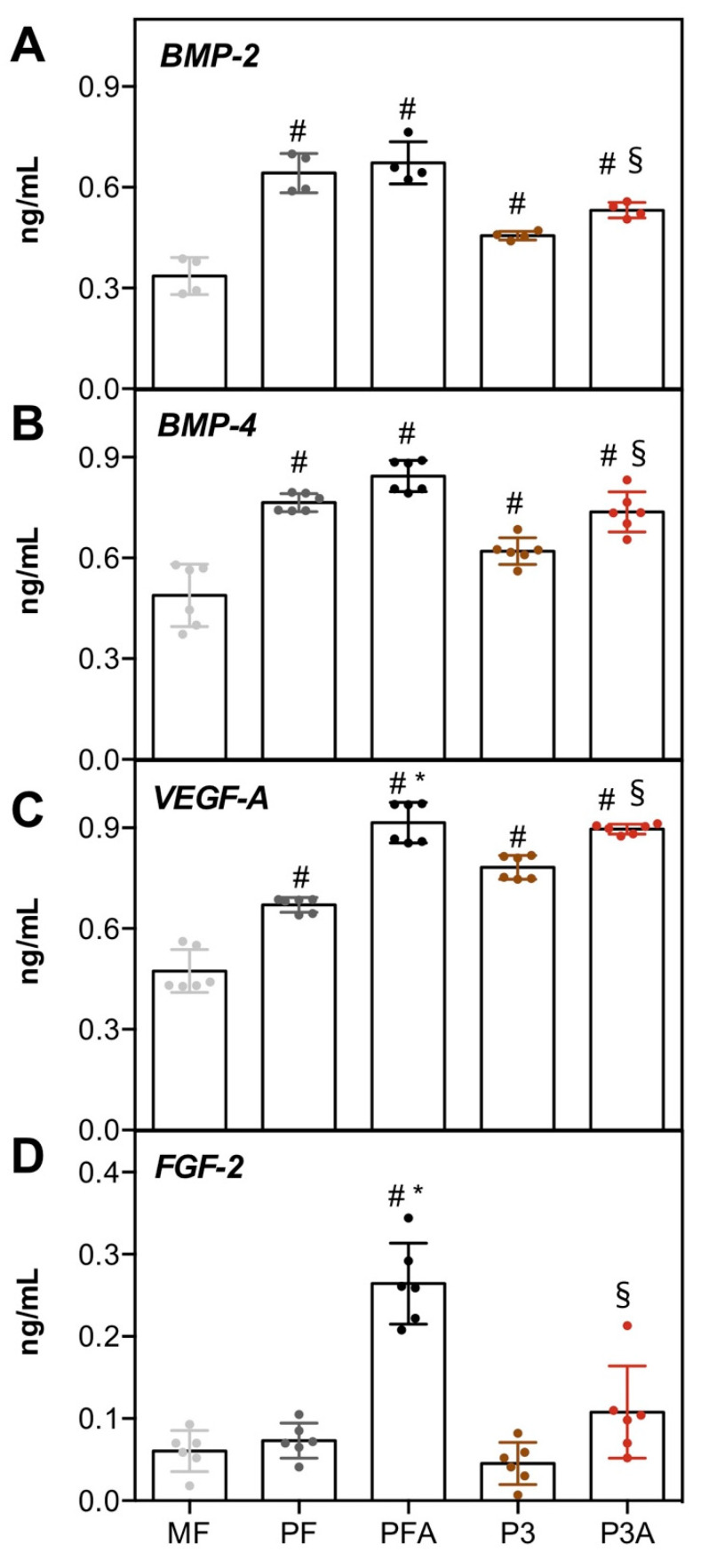
Measurement of BMP-2, BMP-4, FGF-2, and VEGF-A secretion in cell culture medium. hBM-MSCs were seeded on different Ti surfaces, and after 7 days, the cell culture medium was analysed for BMP-2 (**A**), BMP-4 (**B**), VEGF-A (**C**) and FGF-2 (**D**) by ELISA assay. Results (in ng/mL) are expressed as mean ± SD. Statistical significance with respect to MF was indicated by # (MF vs. other samples), while the symbol * indicates differences between PF and PFA (PF vs. PFA), and § between P3 and P3A (P3 vs. P3A).

To investigate whether different titanium topographies may be associated with a different ability of hBM-MSCs to release important bone-related bioactive molecules into the culture media, the secretion of biological components required for osteointegration and osteogenesis, such as BMP-2 (Bone Morphogenetic Protein-2), BMP-4 (Bone Morphogenetic Protein-2), VEGF-A (Vascular endothelial growth factor A) and FGF-2 (Fibroblast Growth Factor-2), was determined over a 7-day period of cell culture (Figure 6). Among the BMPs, BMP-2 and BMP-4 act as master regulators of MSC differentiation into osteoblast and chondrocyte phenotypes, leading to bone and cartilage formation [42]. The levels of BMP-2 and BMP-4 were higher in PF, PFA, P3 and P3A than in MF (# *p* < 0.05). Furthermore, the release of BMP-2 and BMP-4 was significantly increased by acid treatment: higher production of both BMPs was measured in PFA compared to PF (* *p* < 0.05) and in P3A compared to P3 (§ *p* < 0.05) (Figure 6A,B). The same trend was observed for VEGF-4, which is known to be expressed during osteoblastogenesis derived from human mesenchymal stem cells [43], with a crucial role in angiogenesis during bone formation and healing of bone fractures [44]. The level of VEGF-A was significantly higher on 3D-machined surfaces than on MF (# *p* < 0.05), and on those 3D machined with acid (Figure 6C). Finally, the quantification of FGF-2, which represents one of the FGFs mainly expressed at the early stage of osteogenic differentiation [45], revealed that acid surface treatment had an impact on its production, which was higher on PFA than on PF (* *p* < 0.05) and on P3A than on P3 (§ *p* < 0.05, Figure 6D). No changes were observed on 3D-machined surfaces compared to MF (# *p* > 0.05, Figure 6D). Taken together, these data suggest that surface topography, or more precisely the surface achieved by acid treatment, appears to promote osteogenic activation of hBM-MSCs and in part through the release of molecules that signalling pathways are closely involved with in the osteogenic differentiation process.

**Figure 7:**
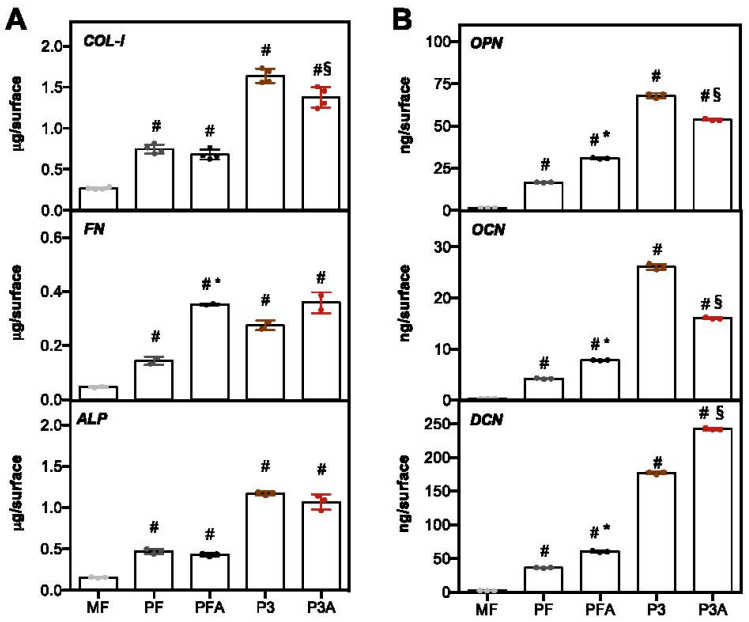
Quantification of bone-ECM protein deposition. Protein quantification of COL-I, FN, ALP, OPN, OCN and DCN produced by hBM-MSCs cultured on different Ti surfaces at day 7 of culture was assessed by ELISA assay as described in Materials and Methods Section. Data are expressed as µg/surface (**A**) and ng/surface (**B**), respectively. Statistical significance with respect to MF was indicated by # (MF vs. other samples), while the symbol * indicates differences between PF and PFA (PF vs. PFA), and § between P3 and P3A (P3 vs. P3A).

During bone regeneration, bone ECM plays an important role in bone formation by dynamically interacting with the main cells involved in this process, osteoblasts, and osteoclasts [46]. Thus, it is a prerequisite for any implant to be able to stimulate bone-ECM formation to drive cellular behaviour, bone tissue function and cohesive integration with the surrounding bone tissue. To further examine the osteogenic activation of hBM-MSCs, an ELISA quantification of bone-ECM proteins was performed (Figure 7). All the proteins analysed represent important markers of bone development: COL-I (type-I collagen) is the main constituent of the ECM organic part [47], whereas OPN (osteopontin) is a glycosylated extracellular phosphoprotein secreted during the early phase of osteogenesis with an important role in cell adhesion and calcification of mineralised tissue [48]. DCN (decorin) is a marker of terminal differentiation of osteoblasts [49], and OCN (osteocalcin) is the most abundant non-collagenous bone matrix protein, often used as a late marker of bone formation [50]. In addition, this protein is also involved in modulating cell–matrix interactions by improving the adhesion of osteoblast-like cells to biomaterials [51]. The results shown in Figure 7 showed a significant increase in all tested ECM proteins in the 3D-printed group compared to the MF control (# *p* < 0.05). However, differences were observed related to surface modification and material structure (solid or porous). A statistically significant increase in the activity of FN (fibronectin), OPN, OCN and DCN can be appreciated for cells cultured on solid 3D-printed samples treated with acid (PFA) compared to PF (* *p* < 0.05, Figure 7A,B). By contrast, higher DCN production was observed in the acid-treated 3D-printed porous sample (P3A) when compared to P3 (§ *p* < 0.05, Figure 7B), while the deposition of COL-I, OPN and OCN was lower in P3A (§ *p* < 0.05, Figure 7A,B). It is also interesting to note that porous 3D-printed implants (P3 and P3A) presented a higher level of COL-I, ALP, OPN, OCN and DCN than flat 3D-printed implants (PF and PFA, *p* < 0.05). Overall, these data are consistent with findings in the literature, demonstrating that modified surface roughness, such as that obtained by acid-etching treatment, can influence the activation of osteoblast differentiation [38,52], providing the starting point for initiating the osseointegration process.

### 2.3. Texture Analysis of SEM Images 

**Figure 8:**
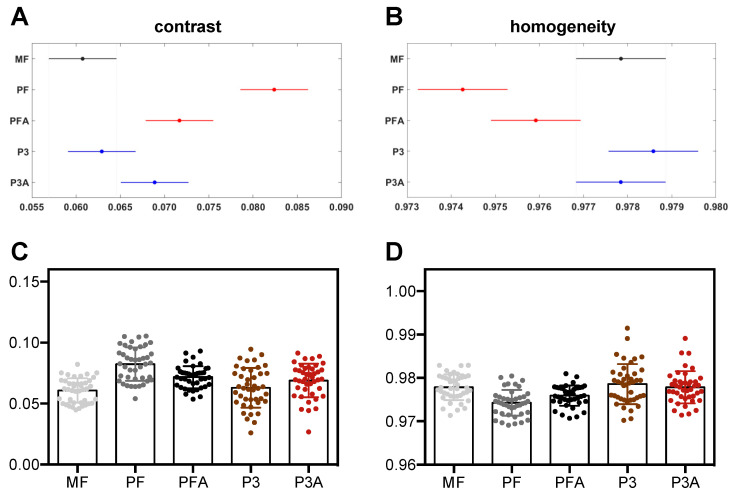
Haralick texture analysis of SEM images. Computation of Haralick’s contrast (**A**) and homogeneity (**B**). The circles represent the means of the contrast or homogeneity, whereas the horizontal bars are the 95%-confidence intervals for the difference between means (i.e., in the comparison of two biomaterials, non-overlapping horizontal bars show statistical significance with *p* < 0.05). Contrast and homogeneity represented as mean value ± SD ((**C**,**D**), respectively).

New advances in regenerative medicine and tissue engineering have been made possible by understanding how biomaterial surfaces can control specific cellular behaviours such as adhesion, growth, differentiation, and migration. Living cells have an intrinsic ability to perceive, integrate and respond to environmental signals at the micro- and nanoscale [53,54]. Topographical features such as roughness and nanoscopic surface pores can impact cellular behaviour [55,56,57]. The wide range of methods used to investigate surface characteristics, such atomic force microscopy and scanning electron microscopy, can provide important information to enhance our understanding of cell behaviour on biomaterials. In this context, we recently reported that nanotopography-induced changes in cell phenotype and proliferation can be predicted via a computer vision approach [58], the so-called analysis of Haralick’s features, where an imaging technique (e.g., SEM) is followed by a texture analysis of the obtained images [59]. Haralick’s features are derived from the grey-level co-occurrence matrix (GLCM) and are utilised in many fields such as land-use and forest-type classification [60], fabric defect recognition [61] and in medicine: e.g., skin texture [62], MRI images of the liver [63], X-ray mammography [64], breast cancer [65], brain cancer [66], tumour phenotype [67], and tumour classification [68]. Here, Haralick’s textural features of unseeded Ti6Al4V surfaces were extracted to correlate the texture features to the observed biological parameters, in particular the expression of osteopontin and osteocalcin of hBM-MSCs seeded onto the different Ti6Al4V surfaces tested. As shown in Figure 8, the MF surface showed, as expected, low contrast and high homogeneity, both related to low expression of osteopontin and osteocalcin reported in Figure 7. In general, 3D printing caused a slight increase in contrast features for the porous group, with statistically significance differences among control and etched P3A, and larger and significant increases for non-porous samples (PF, PFA) compared to the MF control (Figure 8A,C). On the other hand, compared to the MF control, 3D printing did not affect homogeneity in the porous group (P3 and P3A) but did in the non-porous groups (Figure 8B,D). Interestingly, the most statistically significant difference, also with Haralick’s parameters, is among the non-etched 3D-printed materials (P3 and PF). However, this significant distinction is not present for their etched versions (P3A and PFA), which have intermediate and not significantly different values (Figure 8A–D). The previous results can be related to the expression of osteopontin and osteocalcin (Figure 7) where, in 3D-printed biomaterials, a lower value of contrast (or a higher value of homogeneity) predicts a higher value of protein expression.

## 3. Materials and Methods

### 3.1. Implant Preparation and Characterization 

#### 3.1.1. Implant Manufacturing

3D-printed titanium endplate samples were provided by Orthofix Medical Inc. (Lewisville, TX, USA) and analysed in the Characterization Center for Materials and Biology (CCMB) at the University of Texas (Arlington, TX, USA). All samples have same overall dimensions (12 mm × 3 mm round disk) but each sample group represents a unique manufacturing process condition. Table 1 lists the sample groups and their processing conditions. Sample group MF is a machined solid Ti alloy (Ti6Al4V) group, included as a plain control as a representing surface morphological condition used commonly in spinal devices. Sample groups PF and PFA are 3D-printed solid flat disks and both groups underwent the same process except for the acid-etching process: group PFA was acid-etched while group PF was not. Similar to group PF and PFA, two groups of 3D-printed porous Ti6Al4V samples, P3 and P3A, with and without etching, were included in the study. The difference between groups PF and PFA and groups P3 and P3A is that groups P3 and P3A are porous disks with a porosity of 50%, which is identical to that of PTC porous endplates. Except for group MF, which was made at Orthofix’s internal machine shop, all other samples were manufactured at Surface Dynamics LLC., the production vendor for PTC product lines, to ensure the processes are consistent with production PTC parts. The typical pictures of the sample groups are shown in Figure 1A–E.

#### 3.1.2. Investigation of Implant Morphology Surface

The implant surface morphology was examined by scanning electron microscopy (SEM). The microscale surface morphologies were characterised in a Hitachi S-4800 field-emission SEM (Tokyo, Japan) using an accelerating voltage of 20 kV and a working distance of ~5 mm. A magnification of 300× was used for MF, PF and PFA groups and 500× was used for P3 and P3A groups. To investigate the presence of nanoscale features due to acid-etching treatment, the surfaces of the porous non-etched sample (P3) and the porous etched sample (P3A) were characterised in a Hitachi S-4800 field emission SEM at magnifications of 80,000× using an accelerating voltage of 2 kV and a working distance of 3.5 to 5.6 mm. The images were taken for all samples from P3 and P3A implants. For each sample, three representative images were taken at a location approximately as seen in Figure 1K. 

#### 3.1.3. Investigation of Implant Roughness Surface

The implant surface roughness was examined by an optical profiler (Veeco NT9100 optical profilometer, Plainview, NY, USA). An area of 1300 μm × 950 μm was used for each measurement for the groups MF, PF and PFA, while an area of 309 μm × 232 μm was used for group P3 and P3A. Roughness parameters, i.e., root-mean-squared roughness (Rq), roughness average (Ra), average maximum profile height (Rz), maximum profile height (Rt), maximum profile peak height (Rp), maximum profile valley depth (Rv), Skewness (Rsk) and Kurtosis (Rku), were measured from three different locations for each sample. A statistical comparison (Students *T*-Test) was calculated between the MF sample and all other implants in addition to between the acid-etched and non-etched versions of each sample (i.e., PF vs. PFA, and P3 vs. P3A). Significance was set at *p* < 0.05 and indicated with # (MF vs. other samples), * (PF vs. PFA), and § (P3 vs. P3A). 

### 3.2. Biological Investigation

#### 3.2.1. hBM-MSCs Culture and Seeding

hBM-MSCs were isolated and cultured at the University of Pavia (Pavia, Italy), as previously described [69]. The study protocols were approved by the Institutional Review Board of the Fondazione IRCCS Policlinico San Matteo and the University of Pavia (2011). Written informed consent was obtained from all the participants involved in this study. The cells used in all experiments were mainly at passage 4–5 and cultured in mesenchymal stem cell growth media (Lonza, Basel, Switzerland) supplemented with 10% foetal bovine serum, 50 μg/mL penicillin-streptomycin, 1% L-glutamine, and 0.2% fungizone, and cells were incubated at 37 °C, 5% CO_2_ and 100% humidity. Cell seeding density was 10 × 10^4^ cells/implant. Before cell seeding, implants were sterilised in an autoclave for 20 min, at 121 °C and 1 bar of pressure, and then extensively washed with sterile phosphate-buffered saline (PBS) solution.

#### 3.2.2. Cell Viability

As a marker of cell viability, cell mitochondrial activity was evaluated at day 7 of culture with 3-(4,5-dimethylthiazole-2-yl)-2,5-diphenyl tetrazolium bromide assay (MTT; Sigma-Aldrich, St. Louis, MO, USA) as previously described [69]. Aliquots of 100 μL were sampled on a 96-well plate and relative light absorbance was measured at 570 nm with a microplate reader (BioRad Laboratories, Hercules, CA, USA). Titration curve interpolation was used to express the number of cells for each sample.

#### 3.2.3. DNA Content Quantification

Total DNA content in hBM-MSCs seeded on test samples was determined after 7 days of culture. After 7 days of culture, hBM-MSCs were lysed by a freeze–thaw method in sterile deionised distilled water. The released DNA content was evaluated with a fluorometric DNA quantification kit (PicoGreen; Molecular Probes, Eugene, OR, USA), following the manufacturer’s instructions. Briefly, samples were diluted 1:100 in 100 µL of working solution (PicoGreen reagent in Tris-EDTA (TE) buffer, 1:200) for the measurement. Fluorescence was detected in a dedicated 96-well plate at 520 nm, after excitation at 480 nm, with CLARIOstar ^®^ Plus Multi-mode Microplate Reader (BMG Labtech, Ortenberg, Germany). Fluorescence results were interpolated with a previously designed titration curve and expressed as μg of DNA in each condition.

#### 3.2.4. Secreted Protein Quantification

Conditioned media were collected at day 7 and levels of secreted bone morphogenetic protein-2 (BMP-2; Elabscience, Houston, TX, USA), bone morphogenetic protein-4 (BMP-4; Elabscience, Houston, TX, USA), vascular endothelial growth factor A (VEGF-A; Elabscience, Houston, TX, USA), and fibroblast growth factor-2 (FGF-2; Elabscience, Houston, TX, USA) were measured by immunoassay. For each of these proteins, a titration curve was designed with provided standards. Equal volumes (100 μL) of the collected culture media were immobilised on antibody-coated wells and immunoassays of the investigated proteins were developed according to the manufacturer’s instructions. Absorbance of the developed reactions was measured by a microplate reader (BioRad Laboratories, Hercules, CA, USA) at 490 nm. 

#### 3.2.5. ECM Proteins Extraction and ELISA Assay

For the evaluation of extracellular matrix proteins, indirect enzyme-linked immunosorbent assay (ELISA) was performed on test implants at 7 days of culture as previously described [70]. In brief, samples were washed extensively with sterile phosphate buffer and then incubated for 24 h at 37 °C with 0.5 mL of sterile sample buffer (20 mM Tris-HCl, 4 M GuHCl, 10 mM EDTA, 0.066% (*w*/*v*) sodium dodecyl sulphate (SDS), pH 8.0). At the end of the incubation time, the total protein concentration in each sample buffer was determined with a BCA Protein Assay Kit (Pierce Biotechnology, Inc., Rockford, IL, USA) according to the specifications of the manufacturer. Calibration curves to measure alkaline phosphatase (ALP), type-I collagen (COL-I), fibronectin (FN), osteocalcin (OCN), osteopontin (OPN), and DCN (decorin) were prepared in microtiter wells O/N at 4 °C with increasing concentrations of each purified protein (from 10 ng to 2 μg) in coating buffer (50 mM Na_2_CO_3_, pH 9.5). A negative control was prepared with well bottom Bovine serum albumin (BSA) coating. To measure the ECM amount of each protein by ELISA, microtiter wells were coated, overnight at 4 °C, with 100 μL of the previously extracted ECM (1 μg/mL in coating buffer). After three washes with PBS containing 0.1% (*v*/*v*) Tween 20, the wells were blocked by incubating with 200 μL of PBS containing 2% (*w*/*v*) BSA for 2 h at 22 °C. The wells were subsequently incubated for 1.5 h at 22 °C with 100 μL with anti-ALP, anti-COL-I, anti-DCN, anti-OPN, anti-OCN polyclonal antisera (1:1000 dilution in 1% BSA, kindly provided by Dr. Larry W. Fisher [71]) and anti- human FN rabbit polyclonal IgG (1:1000 in 1% BSA). After washing, incubation with 100 μL/well of horseradish peroxidase (HRP)-conjugated goat anti-rabbit IgG (1:1000 dilution in 1% BSA) at 22 °C for 1 h was performed. Reaction development with 100 μL/well HRP chromogenic substrate (TMB; Sigma-Aldrich, St. Louis, MO, USA) was carried out and reactions were stopped by adding 100 μL/well of 0.5 M sulfuric acid (H_2_SO_4_). Absorbances at 450 nm were measured with CLARIOstar ^®^ Plus Multi-mode Microplate Reader (BMG Labtech, Ortenberg, Germany). The optical densities from each sample were plotted against a calibration curve containing known amounts of each protein. An underestimation of the absolute protein deposition is possible because the sample buffer, used for matrix extraction, contained sodium dodecyl sulphate, which may interfere with the protein adsorption during ELISA. The amount of extracellular matrix constituents in the different samples was expressed as µg or ng/surface.

#### 3.2.6. ALP Activity

ALP activity was determined using a colorimetric endpoint assay at day 7 as previously reported [70]. The method measures the conversion of the colourless substrate *s* (*p*NPP) by the enzyme ALP into the yellow product *p*-nitrophenol (*p*NP). The rate of colour change corresponds to the amount of enzyme present in the solution. Briefly, an aliquot (0.5 mL) of 0.3 M *p*NPP (dissolved in glycine buffer, pH 10.5) was added to each scaffold at 37 °C. After incubation, the reaction was stopped by the addition of 50 μL 5 M NaOH. The optical density (OD) reading was performed at 415 nm with a microplate reader (BioRad Laboratories, Hercules, CA, USA) using 100 μL of standard or samples and placed into individual wells on a 96-well plate. Samples were analysed in triplicate and compared with the calibration curve of *p*NP standards. ALP activity was expressed as nmol of *p*NP produced per min per cell (Figure 5) or μmol of *p*NP produced per min per μg of total protein or ALP protein (Figure S1A,B, respectively). ALP protein content was determined by ELISA as described in Section 3.2.5.

#### 3.2.7. Scanning Electron Microscopy of Cell-Cultured Implants

hBM-MSCs morphological observations were performed at culture day 7. Cells grown on test samples were fixed with a 2.5% (*v*/*v*) glutaraldehyde solution (Sigma Aldrich, St. Louis, MO, USA) in 0.1 M sodium cacodylate buffer (pH 7.2) for 1 h at 4 °C and washed with sodium cacodylate buffer. Afterwards, samples were dehydrated at room temperature in an ethanol gradient (25-, 50-, 75- and 96%) and lyophilised for 3 h to obtain complete dehydration. The samples were gold sputter coated under nitrogen to make them electrically conductive prior to microscopy at accelerating voltage of 20 kV. A Zeiss EVO MA10 (Carl Zeiss, Oberkochen, Germany) was used to collect pictures at 1000× and 5000× magnification. 

#### 3.2.8. Immunofluorescence

To visualise cell morphology and F-actin fibre distribution, hBM-MSCs were fixed with paraformaldehyde at room temperature on culture day 7 and permeabilised using 0.1% Triton X-100 for 5 min. Cells were then incubated with phalloidin (Alexa-Fluor-568 phalloidin, Invitrogen, Waltham, MA, USA) for 20 min and nuclei were counterstained with Hoechst 33342 (2 μg/mL, Sigma Aldrich, St. Louis, MO, USA). An Olympus BX51 microscope equipped with a 100 W mercury lamp was used to pick images under the following conditions: excitation/emission ~346/460 for Hoechst 33342 and excitation/emission ~578/600 nm for the fluorescence of Alexa-Fluor-568. Images were recorded with an Olympus MagniFire camera system at 10× and 40× magnification and processed with the Olympus Cell F software. 

### 3.3. Texture Analysis of SEM Images 

For each SEM image (500× magnification) of the materials without cells, at least three regions of interest (ROIs) were randomly selected to measure the grey-level co-occurrence matrix (GLCM). After this step, for each GLCM, two Haralick’s features were calculated: the “contrast” and the “homogeneity” [72]. The contrast computes the amount of dissimilarity inside GLCM, and it is a measure of the local variation in pixel values, whereas the other feature is a measure of the local pixel homogeneity.

### 3.4. Statistical Analysis

All statistical calculations were carried out using GraphPad Prism 6.0 (GraphPad Inc., San Diego, CA, USA). Statistical analysis was performed using Student’s unpaired *t*-test and through one-way analysis of variance (ANOVA), followed by Tukey post hoc, for multiple comparisons (significance level of *p* ≤ 0.05).

## 4. Conclusions

In this study, the acid-etching process used in both the solid and porous titanium plates was able to change the surface morphology of the material by creating macroscopic 3D pores with a microscopic rough surface and producing nanoscale surface features on the porous PTC implant endplates. The results clearly demonstrate the importance of the micro- and nanosurface morphology of the Ti6Al4V in determining the fate of the stem cells. Comparison of acid-treated surfaces with untreated surfaces demonstrated that acid treatment influences the response of hBM-MSCs, and that micro and nanoscale features play a critical role in this process. Acid-treated PTC implant endplates proved to be the best choice in terms of human stem cell proliferation and expression of early osteogenic markers. However, further analysis, both *in vitro* and *in vivo* will be required to confirm the ability of this treatment to enhance PTC implant osseointegration beyond the already enhanced cellular osteogenic effects provided by the three-dimensional nature of the PTC implant endplate.

## Figures and Tables

**Table 1:** Summary of the sample groups analysed in this work.

Group	Material	Manufacturing	Post-Process	Porosity
MF	Ti-6Al-4V	machined	non-etched	solid
PF	Ti-6Al-4V	3D-printed	non-etched	solid
PFA	Ti-6Al-4V	3D-printed	etched	solid
P3	Ti-6Al-4V	3D-printed	non-etched	porous
P3A	Ti-6Al-4V	3D-printed	etched	porous

## Data Availability

The data presented in this study are available on request from the corresponding authors.

## Supplementary Materials

The following supporting information can be downloaded at: https://www.mdpi.com/article/10.3390/ijms23137070/s1.
