# Supplementary material for: Early Osteogenic Marker Expression in hMSCs Cultured onto Acid Etching-Derived Micro- and Nanotopography 3D-Printed Titanium Surfaces
Source: Int J Mol Sci. 2022 Jun 25;23(13):7070. doi: 10.3390/ijms23137070 (PMC9266831; doi:10.3390/ijms23137070)
Supplement: Supplementary file 1 [file ijms-23-07070-s001.zip › ijms-1765217-Supplementary.pdf]

Supplementary Material

Early Osteogenic Marker Expression in hMSCs Cultured onto Acid Etching-Derived Micro- and Nanotopography 3D-printed Titanium Surfaces

Nora Bloise [1,2,*], Erik I. Waldorff [3], Giulia Montagna [1,4], Giovanna Bruni [5], Lorenzo Fassina [4], Samuel Fang [3], Nianli Zhang [3], Jiechao Jiang [6], James T. Ryaby [3] and Livia Visai [1,2,*]

[1] Department of Molecular Medicine (DMM), Center for Health Technologies (CHT), UdR INSTM, University of Pavia, 27100 Pavia, Italy; nora.bloise@unipv.it; livia.visai@unipv.it; giulia.montagna04@universitadipavia.it

[2] Medicina Clinica-Specialistica, UOR5 Laboratorio di Nanotecnologie, ICS Maugeri, IRCCS, 27100 Pavia, Italy; nora.bloise@unipv.it; livia.visai@unipv.it

[3] Research and Product Development, Orthofix Medical, Inc., 3451 Plano Parkway, Lewisville, TX 75056, USA; erikwaldorff@gmail.com (E.I.W.); samuel.fang@gmail.com (S.F.); nianlizhang@orthofix.com (N.Z.); jamesryaby@msn.com (J.T.R.)

[4] Department of Electrical, Computer and Biomedical Engineering, Centre for Health Technologies (CHT), University of Pavia, 27100 Pavia, Italy; lorenzo.fassina@unipv.it

[5] C.S.G.I.-Department of Chemistry, Section of Physical Chemistry, University of Pavia, 27100 Pavia, Italy; giovanna.bruni@unipv.it

[6] Department of Material Science, University of Texas, Arlington, TX 76019, USA; jiangjc@uta.edu

* Correspondence: nora.bloise@unipv.it (N.B.); livia.visai@unipv.it (L.V.)

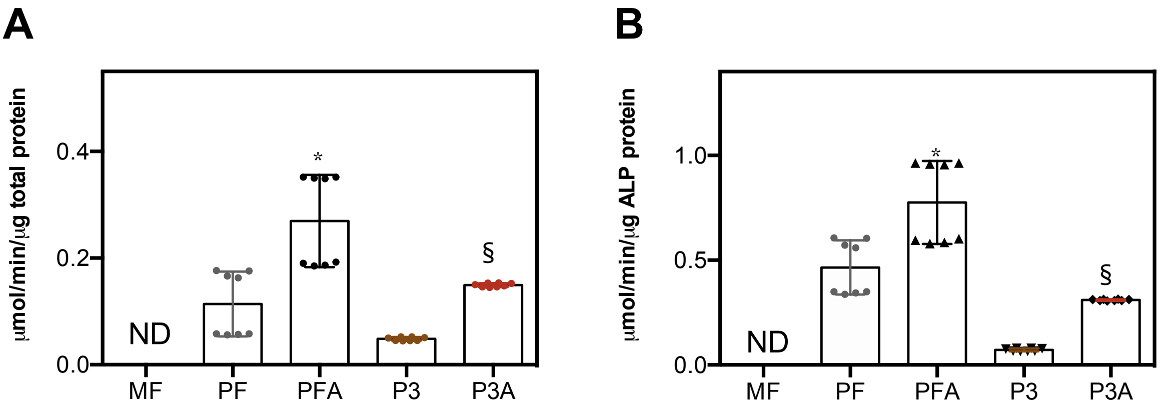

Figure S1 Alkaline phosphatase activity. (A) ALP activity determined colorimetrically, corrected for the protein content measured with the BCA Protein Assay Kit and expressed as micromoles of p-nitrophenol produced per min per ug of total protein (B) ALP activity expressed as micromoles of *p*-nitrophenol produced per min per ug of ALP protein determined by ELISA assay. Statistical analysis was performed by using Student's unpaired t-test (significance level of $p < 0.05$). Symbol * means significant differences between PF and PFA (PF vs PFA) and § between P3 and P3A (P3 vs P3A). ND = Not Detectable.
